# The Expression of miR-34c-5p Induces G0/G1 Cell Cycle Arrest and Apoptosis in SW480 Colon Cancer Cell

**DOI:** 10.5812/ijpr-135501

**Published:** 2023-06-18

**Authors:** Shirin Farzaneh, Shabnam Bandad, Faezeh Shaban, Masoumeh Heshmati, Nooshin Barikrow, Sanaz Pashapour

**Affiliations:** 1 Pharmaceutical Sciences Center, Tehran Medical Sciences, Islamic Azad University, Tehran, Iran; 2 Department of Cellular and Molecular Biology, Faculty of Advanced Science and Technology, Tehran Medical Sciences, Islamic Azad University, Tehran, Iran; 3 Department of Genetics, Faculty of Advanced Science and Technology, Tehran Medical Sciences, Islamic Azad University, Tehran, Iran; 4 Department of Pharmacology and Toxicology, Faculty of Pharmacy and Pharmaceutical Sciences, Tehran Medical Sciences, Islamic Azad University, Tehran, Iran

**Keywords:** Micro RNAs, miR-34c-5p, Tumor Suppression, Colorectal Cancer, Cell Cycle, Apoptosis

## Abstract

**Background:**

Expression of the miR-34 family, including miR-34a/b/c, has been reported to inhibit the progression of several cancer types by inhibiting cell proliferation and inducing apoptosis.

**Objectives:**

We attempted to investigate the effect of SW480 cell transfection with miR-34c-5p mimics on cell proliferation.

**Methods:**

To do this, SW480 colon cancer cell line was transfected with miR-34c-5p mimics, scramble sequence, and the vehicle in PBS mock, and then cell proliferation was assessed by MTT assay. The population of cells in cell cycle phases, ROS generation, and apoptosis rate were evaluated by flow cytometry. Additionally, we determined the relative expression of apoptotic genes through real-time PCR technique.

**Results:**

We observed a reduced proliferation rate in cells transfected with miR-34c-5p compared to the control group (P <0.05). We also found that miR-34c-5p caused a significant increase in apoptosis rate (P < 0.001) and cell cycle arrest in the G0 and G1 phases (P < 0.05). Moreover, a significant increase was reported in the expression of pro-apoptotic genes, including *BAK* (P < 0.001), *BAX* and *BAD* (P < 0.0001), and *Caspase* 7/9 (P < 0.0001).

**Conclusions:**

However, no remarkable difference was seen in the expression of *MCL1*, *BCL2*, and *CASPASE 3* genes. Our conclusion is that overexpression of miR-34c-5p could be considered a promising approach for colorectal cancer treatment.

## 1. Background

Colorectal cancer (CRC) is increasingly becoming a major health problem as the global number of new cases is estimated to reach 3.2 million in 2040, based on the projection of population growth and aging (1-3). Currently, conventional curative options for colon cancer include surgical resection, molecular targeted therapy, and adjuvant radiotherapy or chemotherapy (3-5). Thus, it is important to find novel biomarkers and molecular mechanisms that can be employed in the treatment of colon cancer. Accumulating evidence shows that microRNAs (miRNAs) contribute to the development and progression of various cancers (5). MicroRNAs are small, single-stranded, and noncoding nucleotide sequences that can regulate the expression of specific target genes through translational repression or mRNA degradation (5, 6). MiRNAs are considered potential valuable biomarkers and therapeutic options for cancer, playing a pivotal role in research and clinical settings (7-9). miR-34c-5p is a great example with tumor suppression activity, targeting a myriad of genes related to tumorigenesis and cancer progression (8, 10). Of note, miR-34c-5p can inhibit tumor growth by upregulating apoptotic genes and downregulating the expression of genes associated with cancer cell proliferation (9, 10). Previous studies have reported the downregulation of miR-34c-5p in colorectal cancer, which was inversely correlated with tumor growth and metastasis (10, 11). However, little is known about the mechanism through which the miR-34c-5p exerts its inhibitory effects on colorectal cancer cells.

## 2. Objectives

In this study, we attempted to investigate the effect of miR-34c-5p transfection on cell cycle phases and apoptosis of SW480 colorectal cancer cell lines.

## 3. Methods

### 3.1. Supplying Materials

Dulbecco's modified Eagle medium (DMEM) medium, fetal bovine serum (FBS), trypsin EDTA, phosphate buffered saline (PBS), and penicillin/streptomycin were purchased from Gibco, USA. Dimethyl sulfoxide (DMSO) and DEPC water were obtained from Sigma, Germany. Isopropanol and chloroform were purchased from MERK, Germany, and Trizol LS Reagent solution was provided from Ambion, USA. The cDNA synthesis kit was also purchased from TAKARA, Japan.

### 3.2. Cell Culture and Transfection

To perform cell transfection, Lipofectamine 2000 reagent (Invitrogen) was employed. Cell transfection was done with miR-34c-5p mimics, scramble random sequence (Bioneer), a miRNA without target mRNA, and the vehicle in PBS mock as a negative control. The mature miRNA-34c-5p sequence from the miRbase database has been listed as AGGCAGUGUAGUUAGCUGAUUGC (accession number: MIPF0000039).

### 3.3. Cell Survival Assay

To assess cell survival rate, we established three groups, including miR-34c-5p mimics, scramble, and the vehicle in PBS mock. Upon reaching 70 - 80% confluency, cells were seeded in a 96-well plate at a density of 6 × 103 cells per well and incubated at 37°C for 24 hours. After incubation, SW480 cells transfected with the target groups were incubated for 24, 48, and 72 hours. At the end of the transfection period, the cell culture medium was discarded, and cells were treated with fresh culture medium containing 20 μL tetrazolium salt (MTT, 5 mg/mL). After 4 hours of incubation at 37°C, the medium was replaced with 200 μL DMSO and cells were incubated for 15 min at room temperature in a dark place. Dissolved formazan crystals were quantified by measuring the absorption of samples at 570 nm using an ELISA plate reader (12).

### 3.4. Evaluation of Cell Apoptosis Rate

SW480 cells were plated at a seeding density of 10^4^ × 25 cells/cm^2^ in a T-25 flask. Then cells were transfected with scramble, miR34c-5p mimics, and the vehicle in PBS mock for 48 h. After transfection, cells were double-washed with PBS, detached using trypsin, and centrifuged at 260 RCF for 5 minutes. Next, 0.5 μL of Annexin-V-FLUOS labeled reagent solution, 1 mL of incubation buffer, and 0.5 μL of propidium iodide solution (PI) (Sigma Aldrich, North America) were added to the target groups and incubated for 30 minutes at room temperature in a dark condition. Finally, 700 μL buffer was added to the samples, and the apoptosis rate was determined using flow cytometry (FACSCalibur). The obtained results were then analyzed via FlowJo 7.6.1 (13, 14)

### 3.5. Cell Cycle Assay

Cell cycle assessment was conducted after cell transfection with scrambled miR and miR34c-5p mimics and cell transfection with the vehicle in PBS mock for 48 h. After incubation, the cell pellet of each group was fixed with 70% ethanol. Then PI MASTER MIX solution, including 40 μL PI, 10 μlRNase, and 950 μL PBS, was added to the target groups and incubated at room temperature for 30 min. Cell population in each cell cycle phase was measured and analyzed using FACSCalibur flow cytometry apparatus and FlowJo software (13, 14).

### 3.6. Gene Expression Assessment

SW480 cells were cultured in a T25 cm^2^ flask and transfected with the target groups for 48 hours. Total RNA was manually extracted using Trizol LS Reagent (Ambion, USA). The purity of RNA was measured with a NanoDrop 2000/2000c spectrophotometer (Thermo Fisher Scientific, Wilmington, USA). Reverse transcription of 1 µg total RNA was done using TAKARA cDNA synthesis kit (cat#RP037A). Real-time PCR was conducted using SYBR Green Master Mix (Yekta Tajhiz, Tehran, Iran), with a holding stage at 95°C for 15 minutes and the subsequent 40 cycles of denaturation at 95°C for 15 seconds, annealing at 58 - 60°C for 30 seconds, and extension at 72°C for 30 seconds. Gene expression was analyzed by the 2^-∆∆ct^ Livak method (15). Specific primer sequences have been summarized in Table 1 (16).

**Table 1.** Primer Sequences for Real-time Reverse-transcription PCR Analysis

Gene	Forward and Reverse Primers (5'-3')	PCR Fragment Size (bp)	Length	TM
***B-ACTIN***		66		
F	GGCACCCAGCACAATGAAG		19	59.41
R	CCGATCCACACGGAGTACTT		20	59.18
***CASPASE 3***		140		
F	GAGACAAGCAGCCCTTAGCC		20	60.75
R	GGACAAGCACGGAACAGAG		10	58.47
***CASPASE 7***		101		
F	TCAGAGTTCACGGAGTTCAA		20	56.44
R	ATGTTCTTTCAGCCCCTTTG		20	56.20
***CASPASE 9***		82		
F	TTTGGCAGCAACGACACAGA		20	60.75
R	TGGGTGAGGGGAGCATTACA		20	60.55
***BAX***		166		
F	ACCGCCTCACTCACCATCT		19	60.61
R	GACCACTCTTCCCCACACC		19	59.62
***BAK***		170		
F	ATCAGGGAAAAGGAGTAGGG		20	55.89
R	GAGGGTGGGGGAACAGAG		18	58.60
***BAD***		127		
F	CTCCACATCCCGAAACTCC		19	57.54
R	GTCAGCCCTCCCTCCAAA		18	58.50
***BCL2***		217		
F	CAGACACACACACACACAACAA		22	59.77
R	TTTACAGGCACAGAACATCCA		21	57.50

### 3.7. Statistical Analysis

All data were expressed as mean ± SD of at least three replications. Statistical analysis of the obtained data was performed through two-way analysis of variance (ANOVA) and Tukey's multiple comparison test using GraphPad Prism software. Data with p-values lower than 0.05 were regarded as statistically significant.

## 4. Results

### 4.1. Cell Viability

**Figure 1.**
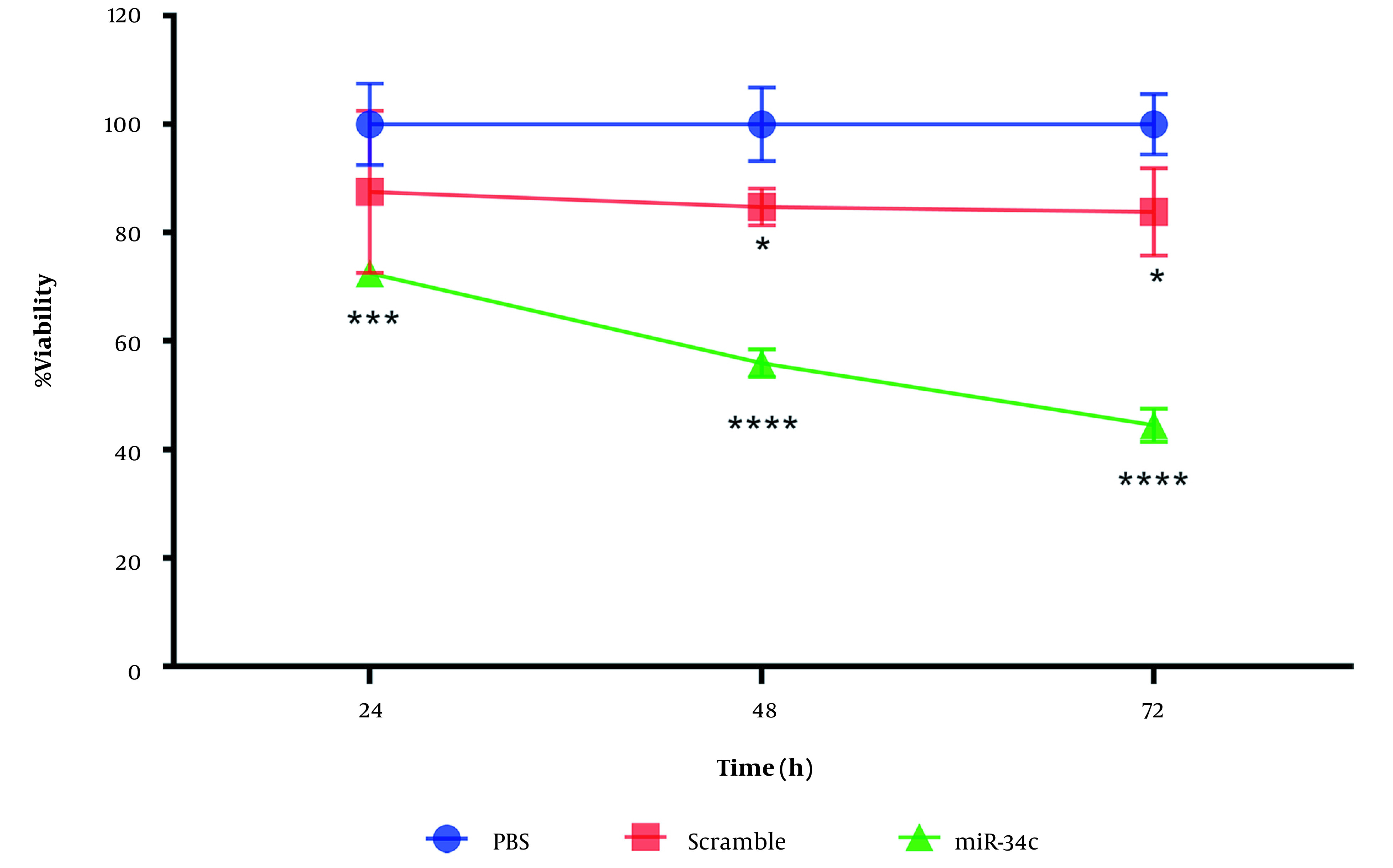
Comparing viability (OD) of SW480 cell line transfected with miR-34c-5p mimics and scramble compared to PBS at three times 24, 48, and 72. *; P < 0.05, ***; P < 0.001, **** and P < 0.0001. Each experiment is performed in triplicate.

We evaluated cell viability percentage through MTT assay. SW480 cell line transfection with miR-34c-5p mimics (20nM), scrambled sequence, and the vehicle in PBS mock as control was performed for 24, 48, and 72 hours with Lipofectamine 2000 (Invitrogen, USA). From Figure 1 it can be seen that in comparison with control cells, miR-34c-5p transfection led to a remarkable decrease in the viability of SW480 cells.

Transfection of the scramble sequence was observed to have no significant effect on SW480 cell viability after 24 h (P = 0.107). However, we found a remarkable decrease in cell survival rate during 48 and 72 h after cell transfection with scramble sequence (P < 0.05). Based on the cell survival assay results, we chose the 48h time span after cell transfection to evaluate apoptosis, cell cycle arrest, intracellular ROS generation, and changes in the expression of the apoptotic and anti-apoptotic genes (Figure 1).

### 4.2. Cell Apoptosis

**Figure 2.**
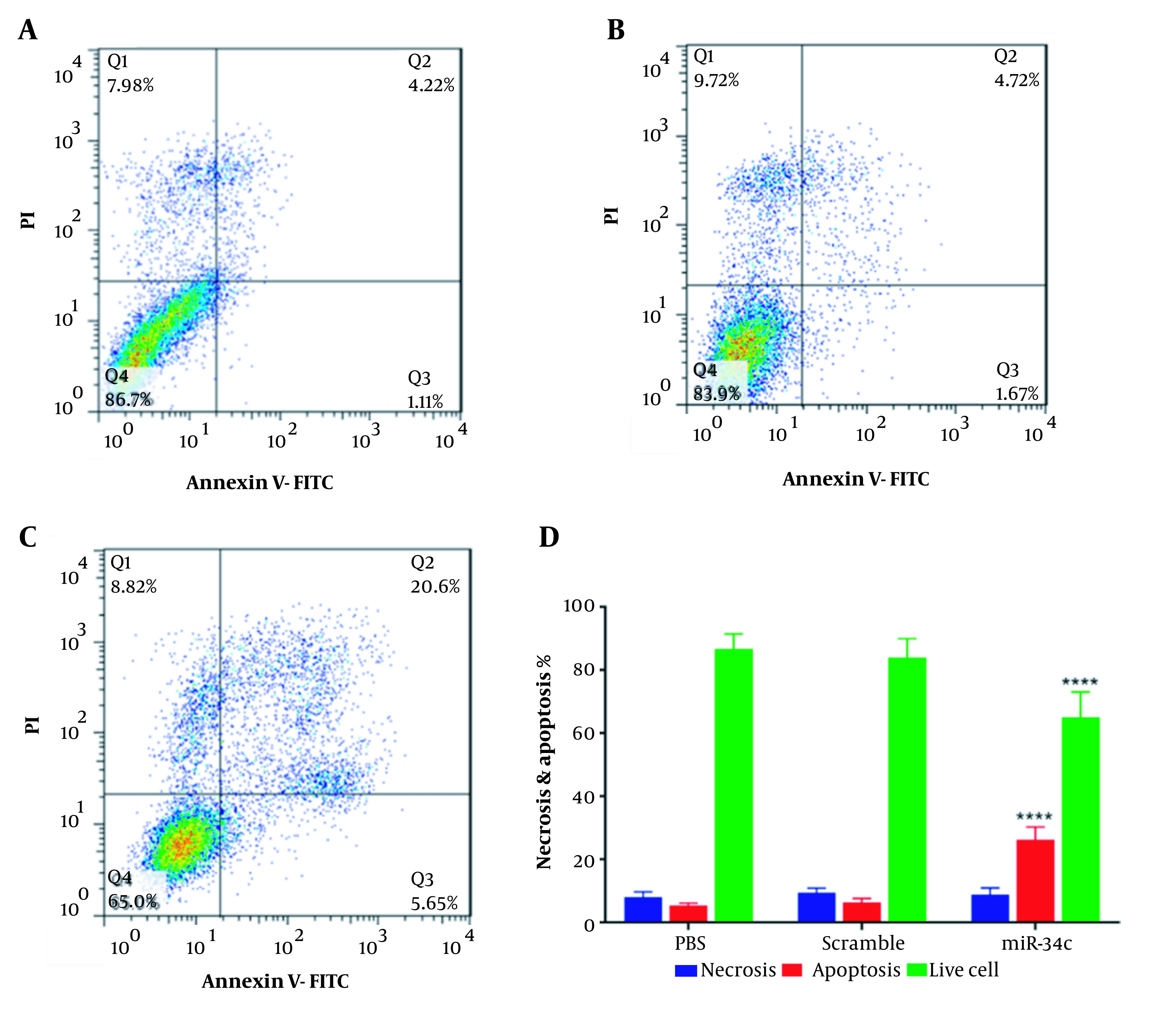
Percentage of cells in various phases of Annexin-V/PI test in SW480 cell line transfected with PBS (A), scramble (B), miR-34c-5p mimics (C), and necrosis and apoptosis rate as well as live cell count in SW480 cells transfected with miR-34c-5p mimics as compared to PBS and scramble groups (D). **** P < 0.0001. Results are shown as representative of three independent experiments. Abbreviations: Q1: An-/pI+ necrosis cells; Q2: marker of delayed apoptotic cells An +/pI +; Q3: marker of primary apoptotic An+/pI-; and Q4: marker of live cells An-/pI-.

Quantitative investigation of apoptosis rate in SW480 cells transfected with the vehicle in PBS mock, scramble sequence, and miR34c-5p mimics is highlighted in Figure 2. It is apparent from Figure 2 that cell transfection with miR-34c-5p could significantly induce apoptosis (26.25 ± 2.3%) as compared with control (5.33 ± 0.462%) and scramble groups (6.39 ± 0.693%, P < 0.0001). Furthermore, there was a remarkable decrease in the percentage of living cells in the miR-34c-5p group (65 ± 6.41%) in comparison with the control (86.7 ± 2.14%) and the scramble groups (83.9 ± 3.46%, P < 0.0001).

Analysis of necrosis percent showed no statistically significant difference in miR-34c-5p-transfected cells and those transfected with the vehicle controls.

### 4.3. Intracellular ROS Generation

**Figure 3.**
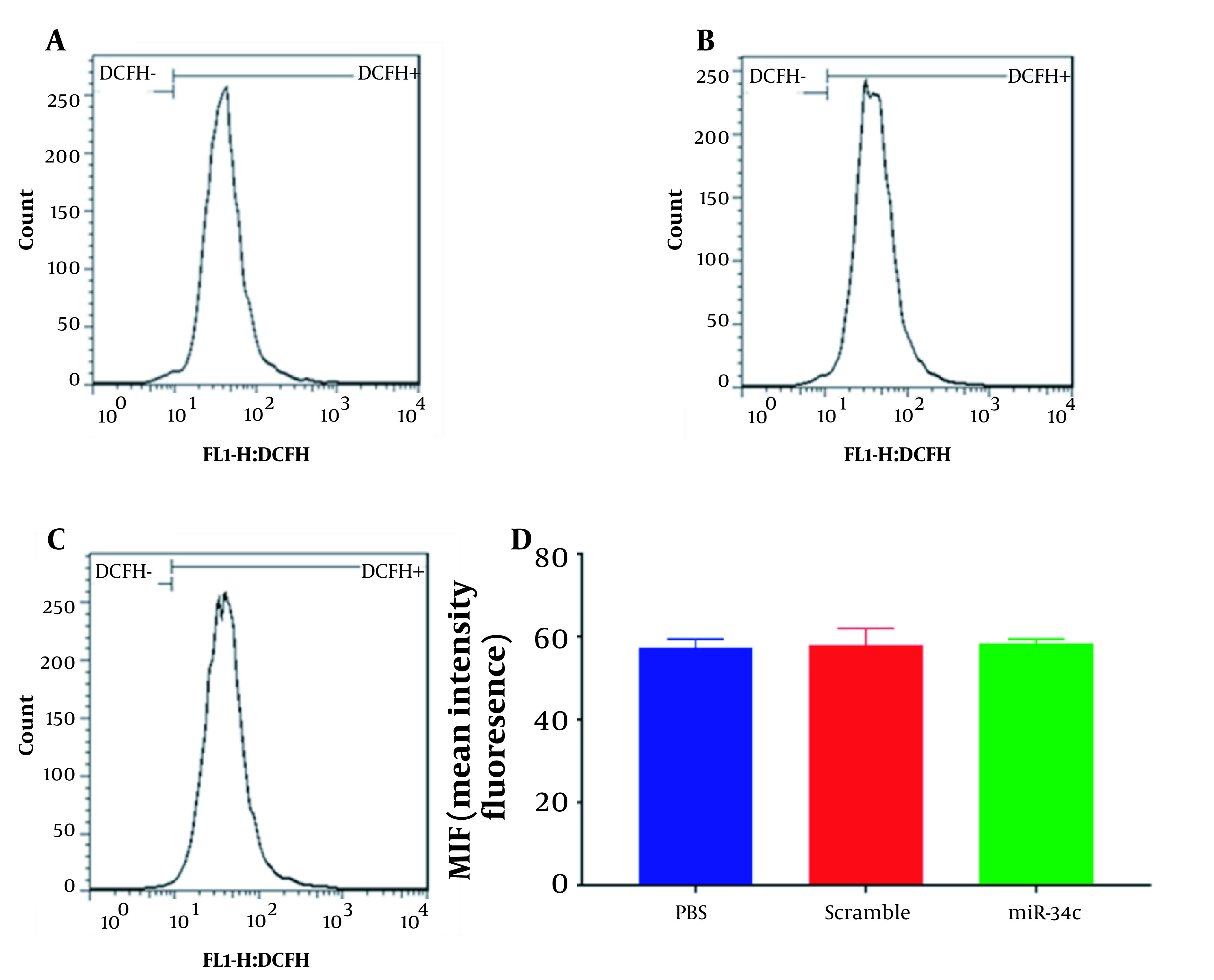
ROS analysis histogram in SW-480 cell line transfected with PBS (A), scramble (B), and miR-34c-5p mimics (C). Comparing ROS formation in SW-480 cell line transfected with miR-34c-5p mimics compared to PBS and scramble (D). Each experiment is performed in triplicate.

SW480 cell line transfection with the vehicle in PBS mock, scramble sequence, and miR-34c-5p mimics was carried out for 48 hours. Then the intracellular ROS was quantified using a flow cytometry instrument. Mean intensity fluorescence was revealed to reach 57.4 ± 1.15, 58 ± 2.3, and 58.4 ± 0.577% in cells transfected with the vehicle in PBS mock, scramble sequence, and miR-34c-5p, respectively (Figure 3). As shown in Figure 3, there was no significant difference in the ROS levels of miR-34c-5p-transfected cells compared to the cells transfected with scramble sequence (P = 0.890) and the vehicle in PBS mock (P = 0.981).

### 4.4. Cell Cycle Analysis

**Figure 4.**
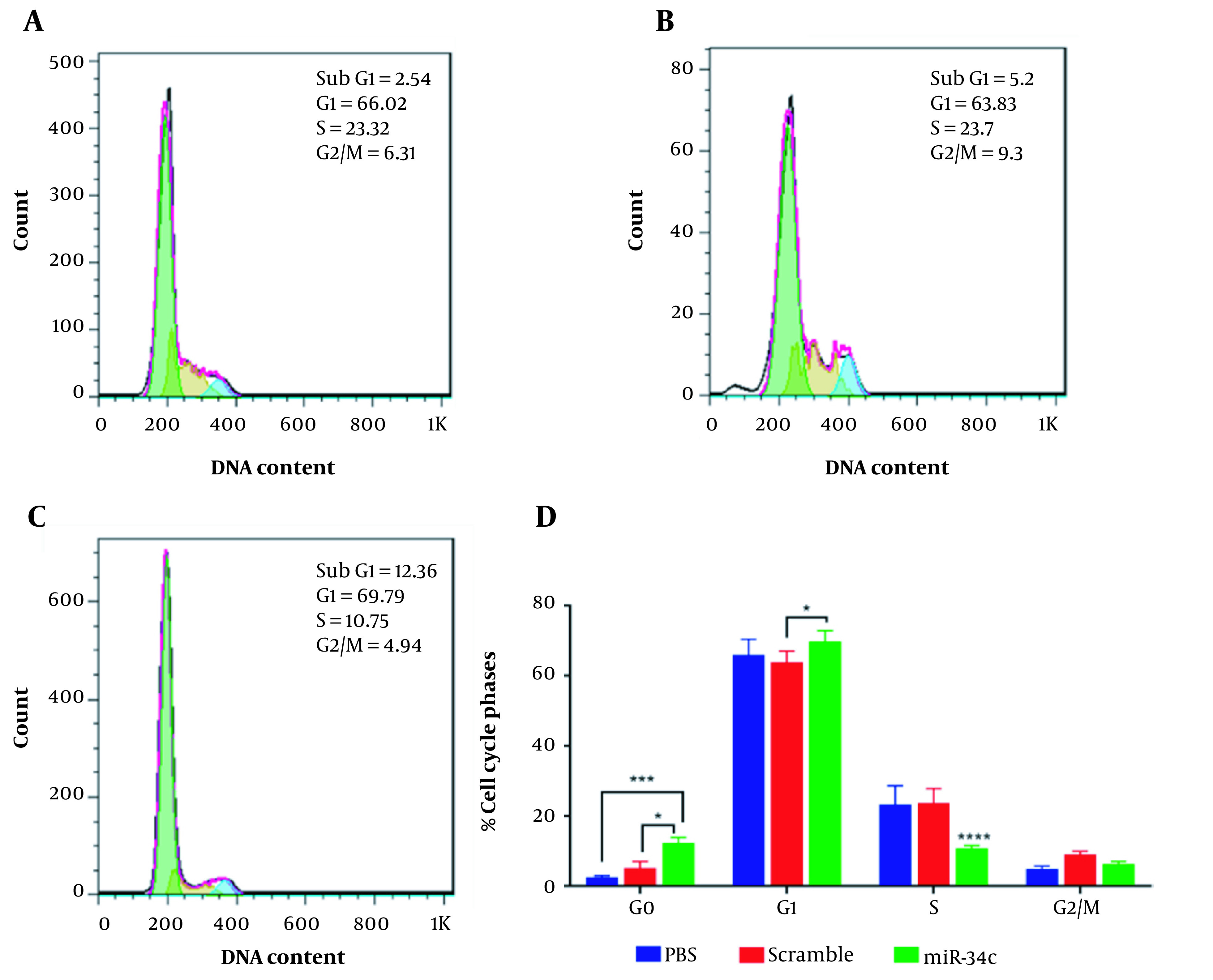
Cell cycle diagram in SW480 cell line transfected with PBS (A), scramble (B), and miR-34c-5p mimics (C). Comparing cell cycle arrest in G0, G1, S, G2/M phases in the treatment of SW480 cell line transfected with PBS, scramble and miR-34c-5p mimics (D). *; P < 0.05, ***; P < 0.001, **** and P < 0.0001. Each experiment is performed in triplicate.

The analysis of SW480 cell population in each cell cycle phase was performed in three scramble, miR-34c-5p mimics, and the vehicle in PBS mock groups using flow cytometry. Figure 4 represents the effect of each transfection on cell cycle arrest in different phases. This figure shows a 4.86-fold and 2.37-fold increase in G0 phase arrest in the miR-34c-5p-transfected cells as compared to the PBS mock group (P < 0.001) and the scramble group, respectively (P < 0.05). It was also revealed that the induction of G1 arrest in the miR-34c-5p-transfected group was 1.09-fold more than the scramble group (P < 0.05) and 1.057-fold more than the PBS mock group (P = 0.238). Furthermore, cell transfection with miR-34c-5p showed that S phase arrest was significantly decreased by 6.79 and 5.25 times compared to PBS mock and scramble, respectively (P < 0.0001). The rate of cell cycle arrest in G2 phase arrest in the miR-34c-5p-transfected group was not significantly different compared to PBS mock (P = 0.818) and scramble groups (P = 0.118). There was also a significant decrease in the expression of miR-34c-5p in cancer cells (data not shown).

### 4.5. Gene Expression Analysis

**Figure 5.**
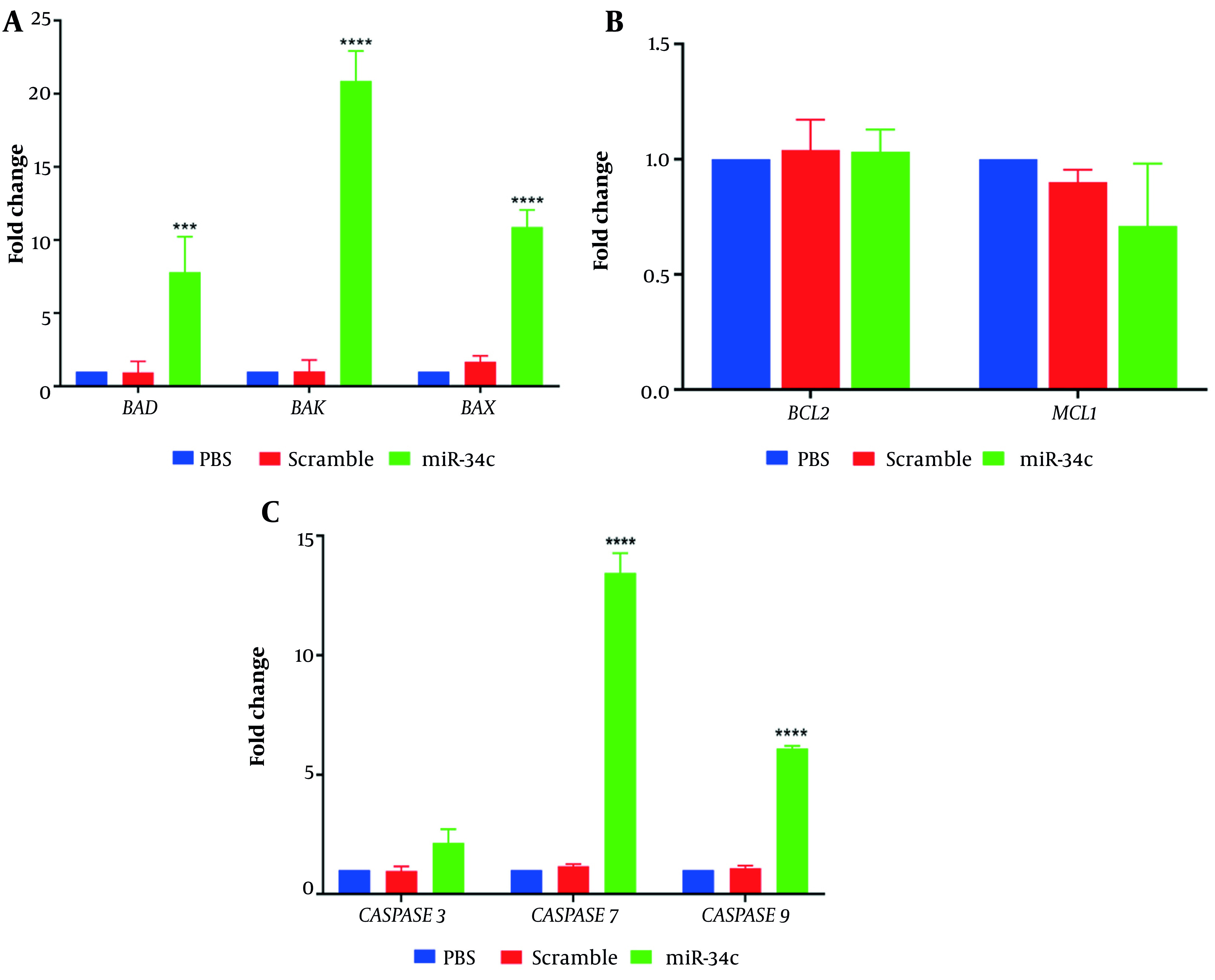
Evaluating the impacts of miR-34c-5p mimics transfection on *BAX*, *BAK*, *BAD* (A), *BCL2*, *MCL1* (B), *CASPASE* 3, 7, 9 (C) gene expression compared to PBS and scramble. ***; P < 0.001 and ****; P < 0.0001. Each experiment is performed in triplicate.

Relative expression of apoptotic and anti-apoptotic genes in SW480 cells transfected with miR-34c-5p mimics, scrambled sequence, and the vehicle in PBS mock was assessed using real-time PCR technique. Figure 5A shows that cell transfection with miR-34c-5p could effectively upregulate the expression of *BAX* and *BAK* pro-apoptotic genes compared to the PBS mock group (P ≤ 0.0001). Additionally, SW480 cells showed increased *BAD* gene expression levels after transfection with miR-34c-5p (P ≤ 0.001). Figure 5B represents the impact of miR-34c-5p transfection on the expression of anti-apoptotic genes. As shown in this figure, there is no significant difference in *BCL2* and *MCL1* gene expression between the miR-34c-5p group and PBS mock (P = 0.436, P = 0.982). Moreover, Caspase 3, 7, and 9 gene expression analysis (Figure 5C) revealed that in cells transfected with miR-34c-5p, there was a significant upregulation in Caspase 7 and 9 gene expression (P ≤ 0.0001), while Caspase 3 expression underwent no remarkable alteration after transfection (P = 0.107).

## 5. Discussion

Aberrant expression of miRNAs may have a dual oncogenic and anti-oncogenic effect on tumor pathology (17). Hitherto, many studies have reported deregulated expression of miRNAs in several cancers (18). In particular, abnormally decreased expression of miR-34 family has been attributed to the prognosis of colorectal cancer (10, 11).miR-34c-5p mimics acts as a tumor suppressor, the downregulation of which can potentially increase the rate of proliferation and metastasis in colon cancer cells (19). The present study showed that miR-34c-5p transfection induced apoptosis, which provoked cell cycle arrest in G1 and G0 phases in the SW480 colon cancer cell line. A possible explanation for this might be the p53 regulatory effects on miRNAs. P53 induces Dorsha and subsequently increases the generation of miRNA (20). The p53 molecule is capable of targeting many cell cycle regulators such as cyclin-dependent kinases 4 and 6 (CDK4, CDK6), Cyclin E2 (CCNE2), and MYC, thereby stimulating the cell cycle arrest in G1 phase (21). This study demonstrated that SW480 cell transfection with miR-34c-5p could effectively induce cell cycle arrest in G0 and G1 and reduce cell proliferation. This finding is consistent with a previous study, which has reported that increased ectopic expression of miR-34c-5p in MDA-MB-231, MDA-MB-468, and BT-549 breast cancer cells induces cell cycle arrest in G2/M phase and decreased *CCND1*, *CDK4*, and *CDK6* gene expression (22). These data further support the tumor suppressive activity of miR-34c-5p in different types of cancers. Another important finding of the current study was that overexpression of *BAD* could inhibit the *BCL2* gene expression, which upregulates *BAX* and *BAK* gene expression.

Moreover, we observed increased expression of *CASPASE 7* and 9 genes, which indicated the induction of the intrinsic apoptosis pathway. Interestingly enough, miR-34c-5p acts as a P53-mediated inducer of apoptosis. In the transfection of M4e cells with lenti-miR-34c-5p, reduced viability rate, apoptosis induction, and increased BCL2 protein levels have been reported (15, 16). Furthermore, a recent study has demonstrated that increased miR-34c-5p expression in NPC cancer cells is associated with reduced cancer cell proliferation, metastasis, and epithelial-mesenchymal transition (EMT) and decreased *BCl2*/*BAX* ratio and *β-CATENIN* gene expression (23). Our findings also agree with the study of Xu et al., who reported that transfection of miR-34c-5p mimic in HK1 and CNE2 cells empowered the expression of *CASPASE* 3, 9 and *BAX* genes and reduced *BCl2* mRNA levels (18). In addition, it has been suggested that overexpression of miR-34c-5p surges the expression of apoptosis-dependent and endoplasmic reticulum genes such as *BAX*, *p-ERK*, *EIF2α*, *IRE1α* and induces ROS generation via the endoplasmic reticulum (19).

Herein, we observed that SW480 cell transfection with miR-34c-5p could decrease cell proliferation and viability. This finding also accords with earlier studies, which reported that miR-34c-5p increases cancer cell sensitivity to chemotherapeutic agents such as cisplatin (21, 24). Further, miR-34c-5p can reduce the number of colon cancer stem cells (CSCs), known as stable cells, which are resistant to conventional therapies and can stimulate tumor regrowth in most cancers after chemotherapy (10, 25). It is also important to note that in a recent study, miR-34a and miR-34b/c gene silencing decreased apoptosis and cell viability and increased tumor grade as well as inducing the expression of lymph node metastasis-associated genes, including *INHBB*, *AXL*, *FGFR1*, and *PDFGRB* (26). Intriguingly, direct prevention of *KITLG* (as a stem cell factor) by miR34c could cause a reduction in the proliferation, migration, and invasion of CRC cells (27).

### 5.1. Conclusions

Our study suggests that overexpression of miR-34c-5p in the SW480 colorectal cancer cell line can induce cell cycle arrest and ROS production and activate apoptosis, thereby diminishing SW480 cell proliferation, invasion, and metastasis. Therefore, miR-34c-5p is a novel and promising option for CRC treatment. However, more research is needed to better understand precise and detailed mechanisms of miR-34c-5p function against tumor growth and spread. Of note, a number of limitations need to be considered. First, the current study was not specifically designed to examine the effect of miR-34c-5p on various colorectal cancer cell lines. Second, this study is limited by the need for more information on the impact of miR-34c-3p on the SW480 cell cycle, viability, and apoptosis, as well as the comparison between miR-34c-5p and miR-34c-3p functions against SW480 cells.

## References

[1] Xi Y, Xu P (2021). Global colorectal cancer burden in 2020 and projections to 2040.. Transl Oncol..

[2] Pashapour S, Heshmati M, Mousavi Z, Esmaeili S (2020). The cytotoxicity of the chloroform and petroleum ether fractional extracts of Galium verum L. in HepG2 and HT29 cell lines.. J Kerman Univ Med Sci..

[3] Krasteva N, Georgieva M (2022). Promising Therapeutic Strategies for Colorectal Cancer Treatment Based on Nanomaterials.. Pharmaceutics..

[4] Petrescu GED, Sabo AA, Torsin LI, Calin GA, Dragomir MP (2019). MicroRNA based theranostics for brain cancer: basic principles.. J Exp Clin Cancer Res..

[5] Schepeler T, Reinert JT, Ostenfeld MS, Christensen LL, Silahtaroglu AN, Dyrskjot L (2008). Diagnostic and prognostic microRNAs in stage II colon cancer.. Cancer Res..

[6] Ardekani AM, Naeini MM (2010). The Role of microRNAs in human diseases.. Avicenna J Med Biotechnol..

[7] Liu J, Yang T, Huang Z, Chen H, Bai Y (2022). Transcriptional regulation of nuclear miRNAs in tumorigenesis (Review).. Int J Mol Med..

[8] Naghizadeh S, Mohammadi A, Duijf PHG, Baradaran B, Safarzadeh E, Cho WC (2020). The role of miR-34 in cancer drug resistance.. J Cell Physiol..

[9] Misso G, Di Martino MT, De Rosa G, Farooqi AA, Lombardi A, Campani V (2014). Mir-34: a new weapon against cancer?. Mol Ther Nucleic Acids..

[10] Roy S, Levi E, Majumdar AP, Sarkar FH (2012). Expression of miR-34 is lost in colon cancer which can be re-expressed by a novel agent CDF.. J Hematol Oncol..

[11] Zhang L, Liao Y, Tang L (2019). MicroRNA-34 family: a potential tumor suppressor and therapeutic candidate in cancer.. J Exp Clin Cancer Res..

[12] Schmittgen TD, Livak KJ (2008). Analyzing real-time PCR data by the comparative C(T) method.. Nat Protoc..

[13] Pashapour S, Heshmati M, Mousavi Z, Esmaeili S (2022). Effect of whole methanolic extract of Galium verum on AGO cell line.. Toxicol Commun..

[14] Pashapour S, Heshmati M, Mousavi Z, Esmaeili S (2022). The apoptotic effect of methanolic extract of Galium verum on HT29 cell line.. J Biol Studies..

[15] Li R, Zhang H, Zheng X (2017). MiR-34c induces apoptosis and inhibits the viability of M4e cells by targeting BCL2.. Oncol Lett..

[16] Rokavec M, Li H, Jiang L, Hermeking H (2014). The p53/miR-34 axis in development and disease.. J Mol Cell Biol..

[17] Baranwal S, Alahari SK (2010). miRNA control of tumor cell invasion and metastasis.. Int J Cancer..

[18] Xu X, Yan H, Zhang L, Liu J, Huang Y, Cheng H (2020). Up-regulation of miR-34c-5p inhibits nasopharyngeal carcinoma cells by mediating NOTCH1.. Biosci Rep..

[19] Tu L, Long X, Song W, Lv Z, Zeng H, Wang T (2019). MiR-34c acts as a tumor suppressor in non-small cell lung cancer by inducing endoplasmic reticulum stress through targeting HMGB1.. Onco Targets Ther..

[20] Sargolzaei J, Etemadi T, Alyasin A (2020). The P53/microRNA network: A potential tumor suppressor with a role in anticancer therapy.. Pharmacol Res..

[21] Hu Y, Yang Q, Wang L, Wang S, Sun F, Xu D (2018). Knockdown of the oncogene lncRNA NEAT1 restores the availability of miR-34c and improves the sensitivity to cisplatin in osteosarcoma.. Biosci Rep..

[22] Achari C, Winslow S, Ceder Y, Larsson C (2014). Expression of miR-34c induces G2/M cell cycle arrest in breast cancer cells.. BMC Cancer..

[23] Wan FZ, Chen KH, Sun YC, Chen XC, Liang RB, Chen L (2020). Exosomes overexpressing miR-34c inhibit malignant behavior and reverse the radioresistance of nasopharyngeal carcinoma.. J Transl Med..

[24] Yang S, Li Z, Luo R (2020). miR-34c targets MET to improve the anti-tumor effect of cisplatin on ovarian cancer.. Onco Targets Ther..

[25] Khan AQ, Ahmed EI, Elareer NR, Junejo K, Steinhoff M, Uddin S (2019). Role of miRNA-regulated cancer stem cells in the pathogenesis of human malignancies.. Cells..

[26] Jiang L, Hermeking H (2017). miR-34a and miR-34b/c suppress intestinal tumorigenesis.. Cancer Res..

[27] Yang S, Li WS, Dong F, Sun HM, Wu B, Tan J (2014). KITLG is a novel target of miR-34c that is associated with the inhibition of growth and invasion in colorectal cancer cells.. J Cell Mol Med..

